# Comparison of Healing Time, Recurrence Rate, Incontinence, and Quality of Life Following Endorectal Advancement Flap versus Cutting Seton Insertion for Trans-Sphincteric High Type Anal Fistula: A 5-Year Retrospective Cohort Study

**DOI:** 10.34172/mejdd.2025.416

**Published:** 2025-04-30

**Authors:** Mohammad Mostafa Safarpour, Khadije Gorgi, Adel Zeinalpour, Sara Shojaei-Zarghani, Zahra Ghanbarzadegan, Seyed Vahid Hosseini

**Affiliations:** ^1^Colorectal Research Center, Shiraz University of Medical Sciences, Shiraz, Iran; ^2^Department of General Surgery, School of Medicine, Shahid Beheshti University of Medical Sciences, Tehran, Iran

**Keywords:** Anal fistulae, Endorectal advancement flap, Cutting Seton insertion, Recurrence rate, Incontinence, Quality of life, Healing time

## Abstract

**Background:**

According to the high prevalence of anal fistulas and the recurrence of the disease following surgery, different methods have been suggested for appropriate treatment of this disease; however, there is no consensus on the most effective method. This study aimed to compare the healing time, recurrence rate, fecal incontinence, and quality of life associated with endorectal advancement flap versus cutting Seton insertion for high-type anal fistulas.

**Methods:**

In this retrospective cohort study, 81 patients with trans-sphincteric high-type perianal fistula, including 53 men and 28 women, were studied for 5 years (2019-2024). The Patients included in this study were divided into two categories. One of them underwent an endorectal advancement flap, and the other one underwent cutting Seton insertion. SPSS software was used for statistical analysis.

**Results:**

37 (45.7%) patients underwent endorectal advancement flap, and 44 (54.3%) patients had cutting Seton insertion. There was no significant difference in recurrence rate, incontinence, and quality of life between the two groups, while the healing time in the group that underwent endorectal advancement flap was significantly different and shorter in comparison with the patients who underwent cutting Seton insertion (30 versus 60 days, respectively, *P*=0.016).

**Conclusion:**

Our study results showed a more significant reduction in the healing time with endorectal advancement flap surgery than the cutting Seton insertion procedure in patients who suffered from trans-sphincteric high-type anal fistula without any significant difference regarding the recurrence rate, incontinence, and quality of life.

## Introduction

 The prevalence of anal fistula as a common disease is 5.6 and 12.3 per 100 000 people in females and males, respectively.^1^ An anal fistula is an abnormal connection between two epithelialized surfaces, typically the anal canal and the surrounding skin. Recurrent pussy discharge or abscess formation is representative of the chronic stage of the infectious process in the anal fistula.^2^ The cryptic glands pussy discharges, and infection near the anus penetrate the skin surface via a tract behind it.^1^ Depending on the fistula tract position relative to the anal sphincter, there are two types of anal fistulas categorized as simple and complex ones. Simple fistulas contain intersphincteric or low transsphincteric ones, and the management will be performed through the opening of the tract. Most fistulas are of this kind.^3^ Complex fistulas include high transsphincteric, suprasphincteric, extrasphincteric, and recurrent fistulas, which present challenging treatment options.^4^ Various surgical methods are available for high-type anal fistula treatment; however, there is no universally accepted best method.^5^ Anal fistula recurrence and fecal incontinence are common and potentially devastating outcomes after anal fistula surgery.^6^

 Cutting Seton insertion and endorectal advancement flap are among the common surgical procedures for high-type anal fistula because of the lower risk associated with recurrence of disease and fecal and gas incontinence.^4^ Seton is made up of indigestible material that is inserted into the fistula tract and gradually removes the sphincter as a drain.^7^ It is thought that the Seton prevents the separation of the sphincter muscle by causing fibrosis and minimizes fecal incontinence. A cutting Seton was placed in the track around the sphincter and tightened at various time intervals as needed till the transection of the sphincter mechanism.^8^ The success rate of the cutting Seton insertion is reported to be about 80%-100%; however, prolonged stool incontinence can be a complication of this procedure.^9^ Another treatment for anal fistula is the mucosal advancement flap.^10^ It is used especially where the sphincter damage is certain. The recurrence rate of this method is reported to be between 8% to 40%.^11^ In this procedure, the preferred method is suturing the internal orifice, opening of the external orifice and eliminating all the contaminated tissues within the fistula. Therefore, the complex fistula can be treated without sphincter damage. Fecal incontinence and the recurrence of perianal fistula in spite of the development of this method are the main challenges of the procedure.^12^ Our study addressed the effect of cutting Seton insertion and endorectal advancement flap on recurrence rate, incontinence, healing time, and quality of life and compared them with global figures.

## Materials and Methods

 In this retrospective cohort study, patients with trans-sphincteric high-type perianal fistula who underwent cutting Seton insertion or endorectal advancement flap procedures performed by the same surgeon (SVH) at Shahid Faghihi and Ghadir Mother and Child Hospitals affiliated with Shiraz University of Medical Sciences, Shiraz, Iran, from January 2019 to January 2024, were included.

 The inclusion criteria for this study were as follows: Suffering from trans-sphincteric high-type perianal fistula, the ability of the patients to provide written informed consent, the age range between 18 and 60 years, and a history of undergoing cutting Seton insertion or endorectal advancement flap surgeries within the past 5 years at the aforementioned medical centers. Suffering from inflammatory bowel diseases, diabetes mellitus, cytomegalovirus infection, malignancy, neurological disorders, previous chemotherapy or radiotherapy, history of previous anorectal surgery due to rectal cancer, history of perianal disease, changing Seton or using another procedure following the first surgery, and using steroids were the exclusion criteria.

 The fistula diagnosis was performed via physical examination by the expert colorectal surgeon in our centers. All patients had soft drinks the night before the operation and received laxatives. The endorectal advancement flap procedure was performed while the patient was placed in a prone position after spinal anesthesia. By injecting methylene blue via the external orifice, the fistula tract, and the internal orifice were detected. Then, an appropriate probe was passed through the external orifice into the tract. The epinephrine solution was injected at a ratio of 1: 200 000 to the submucosal of the flap area. The U-shaped flap was made by the elevation of the mucosa and submucosal of the rectum at the site of the internal orifice. The diseased area was removed, and the site of the internal orifice was sutured and closed with PDS 3-0. Then, the site of the internal orifice was covered with the flap sutured to the area with vicryl 3-0. The external tract fistulectomy was performed to the extent of the sphincter muscles. The site of operation was controlled for hemostasis, and a sterile dressing was applied. The procedure of the cutting Seton insertion was as follows: After spinal anesthesia, the patient was placed in the prone position. After injecting the methylene blue into the external orifice, the fistula tract, and the internal orifice were determined. Then, an appropriate probe was passed. An elastic Seton was inserted into the tract under the guide of the probe and was tightened with silk 2-0 to provide a sustained continuous gentle pressure. Then, the skin and the subcutaneous tissue over the tract were cut and opened with electrocautery. The external tract fistulectomy was performed to the extent of the sphincters. The site of operation was controlled for hemostasis, and a sterile dressing was applied. Post-operation, the patients who underwent endorectal advancement flap were Nothing by Mouth (NPO) for three days and received intravenous antibiotics, including ceftriaxone, metronidazole, and oral diphenoxylate, to prevent fecal excretion. Then, they were discharged with oral antibiotics, and a regular diet was started. After the surgery, the patients who underwent cutting Seton insertion received intravenous antibiotics, ceftriaxone, and metronidazole. The diet was started after recovery. They were discharged the day after the operation. All the patients came to the clinic in determined time, and routine follow-up was performed with caution about the tightening of the cutting Seton every 2 weeks if needed. It should be mentioned that in an infected high-type fistula with pussy discharge or abscess formation, an endorectal advancement flap is not a suitable procedure. Also, in some cases, the internal orifice cannot be identified; therefore, an endorectal advancement flap is not appropriate.

 Medical records were used to identify eligible patients. The information that was obtained from the patients’ medical files consisted of age, sex, marital status, education, date of the surgery, body mass index, smoking, alcohol consumption, and duration of the operation. Our outcomes, including healing time, recurrence rate, incontinence, and quality of life, were collected by telephone. The fistula was considered to be healed if the external opening was closed and there was no pain or discharge from the region for more than 3 months. The time to healing was recorded. Recurrence was considered when the patient had reappearance of the symptoms after the fistula was healed.^13^ A colorectal surgeon conducted a physical examination to confirm recurrence in patients who consented to visit our clinic. Incontinence to solid, liquid, or gas, the need to wear pads and lifestyle alterations were evaluated via the Wexner questionnaire. Each item included five levels of severity (from 0 to 4, meaning never to always). Higher scores indicated severe fecal incontinence.^14^ Quality of life was assessed using the Short Form 12 Health Survey (SF-12) questionnaire, which includes eight health concepts: general health, physical functioning, role-physical, bodily pain, vitality, social functioning, role-emotional, and mental health. This tool is hypothesized to form two dimensions: a physical component summary and a mental component summary.^15^ The validity and reliability of these questionnaires have been assessed previously.^16,17^

###  Statistical Analysis

 The statistical analyses were performed utilizing IBM SPSS Statistics version 25. The assessment of normality for quantitative data was carried out through the Kolmogorov-Smirnov test, followed by the application of either an independent sample t-test or Mann-Whitney U test, depending on the normality status of the data, to compare outcomes between two groups. Additionally, chi-square or Fisher’s exact tests were employed to compare qualitative variables across two groups. *P* value < 0.05 was considered significant.

## Results

 Among the available medical records (n = 118), eight patients were excluded due to corticosteroid use, four patients due to cancer, 14 patients were not willing to participate, nine were non-Persian, and two were deceased (17 from the cutting Seton group and 20 from the endorectal advancement flap group were excluded). Finally, 81 eligible patients (65.4% male), comprising 37 (45.7%) endorectal advancement flap and 44 (54.3%) cutting Seton insertion, with a mean age of 44.49 ( ± 10.97) were included in the study. The median duration of follow-up was 30.00 months (interquartile range [IQR]: 16.00-55.00).

 The demographic characteristics of the patients included in this study are shown in Table 1. There was no significant difference between the two groups regarding sex (*P* = 0.570), ethnicity (*P* = 0.839), marital status (*P* = 0.235), education (*P* = 0.784), and age at surgery (*P* = 0.068). None of the patients had a history of hysterectomy. There was also no significant difference between the two groups regarding total number of delivery (2.00 [IQR: 1.00-4.00] in the endorectal advancement flap versus 2.50 [IQR: 2.00-3.75] in the cutting Seton insertion group, *P* = 0.829) as well as number of women with natural vaginal delivery (4 in the endorectal advancement flap versus 2 in the cutting Seton insertion group, *P* = 0.638).

**Table 1:** Comparison of demographic characteristics between the groups

**Variable**	**ERF**	**Cutting Seton insertion**	***P*** ** value**
n (%)	37 (45.7)	44 (54.3)	
Sex, n (%)			
Male	23 (62.2)	30 (68.2)	0.570^†^
Female	14 (37.8)	14 (31.8)	
Ethnicity, n (%)			
Fars	26 (70.3)	30 (68.2)	0.839^†^
Others	11 (29.7)	14 (31.8)	
Marital status, n (%)			
Single	8 (21.6)	7 (15.9)	0.235^‡^
Married	27 (73.0)	37 (84.1)	
Widowed/Divorced	2 (5.4)	0 (0.0)	
Education, n (%)			
Elementary school	4 (10.8)	7 (15.9)	0.784^†^
Middle school	6 (16.2)	9 (20.5)	
High school diploma	8 (21.6)	10 (22.7)	
Academic degree	19 (51.4)	18 (40.9)	
Age at questionnaire completion (years), mean ± SD	42.65 ± 11.15	46.07 ± 10.70	0.166^*^
Age at surgery (years), mean ± SD	39.62 ± 10.96	44.11 ± 10.72	0.068^*^

Abbreviations: ERF: endorectal advancement flap; SD: standard deviation Between-group differences in variables were determined using an independent sample t-test (*) for parametric variables and chi-square test (†) or Fisher’s exact test (‡) for categorical variables.

 When comparing behavioral and medical conditions between groups (Table 2), no significant differences were found except for a history of chronic constipation (*P* = 0.049) and time of discharge after surgery (*P* < 0.001). Patients who underwent endorectal advancement flap surgery had a significantly higher frequency of a previous history of chronic constipation and longer hospital stays compared with the other group.

**Table 2:** Comparison of behavioral and medical features between the two groups

**Variable**	**ERF**	**Cutting Seton insertion**	***P*** ** value**
Chili pepper consumption, n (%)			
Never/Seldom	28 (75.7)	32 (72.7)	0.763^†^
Yes	9 (24.3)	12 (27.3)	
Current daily tobacco use, n (%)	13 (35.1)	13 (29.5)	0.591^†^
Alcohol intake, n (%)	4 (10.8)	4 (9.1)	> 0.999^‡^
History of chronic constipation, n (%)	15 (40.5)	9 (20.5)	**0.049** ^†^
History of chronic diarrhea, n (%)	2 (5.4)	1 (2.3)	0.590^‡^
History of hard stool, n (%)	1 (2.7)	7 (15.9)	0.065^‡^
Number of previous surgeries for anal fistula, n (%)			
0	25 (75.7)	31 (70.5)	0.845^†^
1-2	6 (16.2)	8 (18.2)	
≥ 3	3 (8.1)	5 (11.4)	
Time of sitting on the toilet, n (%)			
< 3 minutes	17 (45.9)	23 (52.3)	0.584^†^
3-10 minutes	15 (40.5)	18 (40.9)	
> 10 minutes	5 (13.5)	3 (6.8)	
Fecal incontinence, n (%)			
No/Mild	26 (70.3)	32 (72.7)	0.807^†^
Moderate/Severe	11 (29.7)	12 (27.3)	
Number of bowel movements/week, median (IQR)	14.00 (7.00-19.25)	7.00 (7.00-14.00)	0.571^¶^
Hospital discharge after surgery (day), median (IQR)	4.00 (4.00-5.00)	2.00 (1.00-3.00)	**<0.001** ^‡^
Body mass index, mean ± SD	27.48 ± 5.04	28.09 ± 4.41	0.566^*^

Abbreviations: ERF: endorectal advancement flap; IQR: interquartile range, SD: standard deviation. Between-group differences were determined using an independent sample t-test for parametric variables (*), Mann Whitney U test for non-parametric parameters (¶), and Chi-square (†) or Fisher exact (‡) tests for categorical variables. Bold denotes statistical significance (*P* < 0.05).

 In the present study, a quicker healing process was observed in patients who underwent endorectal advancement flaps than those who underwent cutting Seton insertion (*P* = 0.016). However, there were no significant differences between the two groups regarding recurrence rate, incontinence, and quality of life (Table 3).

**Table 3:** Comparison of healing, recurrence, and quality of life between the two groups

**Variable**	**ERF**	**Cutting Seton insertion**	***P*** ** value**
Healing, n (%)	30 (81.1)	34 (77.3)	0.675^†^
Healing time (day), median (IQR)	30.00 (19.50-37.50)	60.00 (27.75-90.00)	**0.016** ^¶^
Recurrence, n (%)	9 (24.3)	12 (27.3)	0.763^†^
Physical component score, median (IQR)	56.58 (51.96-57.38)	54.46 (48.08-56.76)	0.074^¶^
Mental component score, median (IQR)	53.74 (42.60-56.38)	52.70 (40.05-55.90)	0.510^¶^

Abbreviations: ERF: endorectal advancement flap; IQR: interquartile range, SD: standard deviation. Between-group differences were determined using the Mann-Whitney U test for non-parametric parameters (¶) and the Chi-square (†) test for categorical variables. Bold denotes statistical significance (*P* < 0.05).

## Discussion

 According to the high prevalence of the anal fistula, postoperative probable disabling complications, and recurrence rate, different methods of treatment have been investigated to identify a procedure with the least injury to the external sphincter. Endorectal advancement flap and cutting Seton insertion are among the most widespread methods of treating high-type fistulas. In our study, the comparison between these two procedures was performed in patients who suffered from high-type fistulas, considering the postoperative recurrence rate, quality of life, incontinence, and healing time.

 According to our study, the healing process in patients who underwent endorectal advancement flap was quicker and shorter than when patients underwent cutting Seton insertion. According to Jarrar and colleagues, almost all perianal and anovaginal fistulas can be healed with an endorectal advancement flap with the correct identification of the anatomy of the fistula, adequate simplification of the flap, careful technique, and appropriate follow-up.^18^ In concordance with our study, Mohammed and others described a statistically significant difference between the endorectal advancement flap and cutting Seton insertion regarding the healing time with a mean period of 30 versus 77 days, respectively.^19^ However, in the Patton and colleagues study, the mean healing time was 17.7 months (range 3-94 months).^20^

 According to the results of our study, the postoperative recurrence rate of anal fistula had no statistically significant difference between both groups (24.3% in endorectal advancement flap versus 27.3% in cutting Seton insertion). Ghahremani and others (2012) explained that the endorectal advancement flap was a suitable method for the treatment of perianal fistula.^21^ The low recurrence rate of this procedure can be due to the external orifice drainage and the closure of the internal orifice.^22^ the points that were considered in our study, too. In the study of Ege and others, the recurrence rate in the cutting Seton insertion technique was lower than that of the endorectal advancement flap, which may be due to differences in the type of the Seton material.^23^ Also, Van der Hagen et al reported that the recurrence rate of the disease post endorectal advancement flap was higher than the cutting Seton insertion procedure.^11^ In the study by Buchanan et al, the recurrence rate after endorectal advancement flap increased due to the patients’ postoperative pain and spasm, decreased perfusion, and flap tissue necrosis.^24^ Recurrence in both endorectal advancement flap and cutting Seton insertion can be the result of the surgeon’s insufficient experience and failure to diagnose the internal orifice, horseshoe fistula, nicotine administration, and the history of chronic constipation.^21,25^ In addition, the recurrence of the cutting Seton insertion procedure depends on the type of Seton material, lack of inadequate drainage from the internal orifice, and discharge from the external orifice.^25^

 In our study, there was a clinical difference between the two groups regarding mild fecal incontinence postoperatively, which was higher in cutting Seton insertion (32 versus 26 patients in cutting Seton and endorectal advancement flap groups, respectively) but no statistically significant difference in postoperative rate of fecal incontinence was seen between both groups. However, Buchanan et al reported a lower rate of fecal incontinence in the rectal advancement flap than that of the cutting Seton insertion method.^24^ Also, in some other studies, the rate of fecal incontinence was lower in patients who underwent endorectal advancement flap than in those who underwent cutting Seton insertion.^17,23^

 Based on our study, no statistically significant difference was seen between the two groups regarding the quality of life. Quality of life and patient satisfaction are two important items when evaluating any treatment method. In Grucela et al study, overall, most patients had quality of life improvement after undergoing anorectal surgery, and with regard to the anal fistula, the patients had an improved quality of life without identifying any specific procedure.^26^ According to Mylonakis et al post-surgical treatment of anal fistula, the quality of life of the patients remains satisfactory if we encounter simple fistula without any secondary tracks or unidentifiable primary opening.^18^

 The major limitations of our study were the relatively small number of patients, the lack of access to some patients for physical examination in order to detect recurrence, and the retrospective single-center study. Also, we should consider the different methods of assessing postoperative complications, the great heterogeneity in the definition of the type of fistula, the failure to clarify the extent of fistulotomy, and even the characterization of fecal incontinence as the limitations of our study.

## Conclusion

 According to this study, there was no significant difference between the recurrence rate, incontinence, and quality of life between the patients who underwent endorectal advancement flap and cutting Seton insertion, while a statistically significant difference was identified between the two groups regarding the healing time, which was shorter in endorectal advancement flap group. Therefore, it is important to individualize the selection of the suitable procedure for the surgical treatment of the anal fistula and choose the best method according to the experience of the surgeon, postoperative convenience of the patient and return to routine life, previous anal diseases, and sphincter damage.
